# Act In case of Depression: The evaluation of a care program to improve the detection and treatment of depression in nursing homes. Study Protocol

**DOI:** 10.1186/1471-244X-11-91

**Published:** 2011-05-20

**Authors:** Debby L Gerritsen, Martin Smalbrugge, Steven Teerenstra, Ruslan Leontjevas, Eddy M Adang, Myrra JFJ Vernooij-Dassen, Els Derksen, Raymond TCM Koopmans

**Affiliations:** 1 Department of Primary and Community Care, Center for Family Medicine, Geriatric Care and Public health, Radboud University Nijmegen Medical Centre, the Netherlands; 2 Department of Nursing Home Medicine, EMGO Institute for Health and Care Research, VU University Medical Center, Amsterdam, the Netherlands; 3 Department of Epidemiology, Biostatistics, and HTA, Radboud University Nijmegen Medical Centre, the Netherlands; 4 Scientific Institute for Quality of Healthcare, Radboud University Nijmegen Medical Centre, the Netherlands; 5 Kalorama Foundation, Beek-Ubbergen, the Netherlands

## Abstract

**Background:**

The aim of this study is evaluating the (cost-) effectiveness of a multidisciplinary, evidence based care program to improve the management of depression in nursing home residents of somatic and dementia special care units. The care program is an evidence based standardization of the management of depression, including standardized use of measurement instruments and diagnostical methods, and protocolized psychosocial, psychological and pharmacological treatment.

**Methods/Design:**

In a 19-month longitudinal controlled study using a stepped wedge design, 14 somatic and 14 dementia special care units will implement the care program. All residents who give informed consent on the participating units will be included. Primary outcomes are the frequency of depression on the units and quality of life of residents on the units. The effect of the care program will be estimated using multilevel regression analysis. Secondary outcomes include accuracy of depression-detection in usual care, prevalence of depression-diagnosis in the intervention group, and response to treatment of depressed residents. An economic evaluation from a health care perspective will also be carried out.

**Discussion:**

The care program is expected to be effective in reducing the frequency of depression and in increasing the quality of life of residents. The study will further provide insight in the cost-effectiveness of the care program.

**Trial registration:**

Netherlands Trial Register (NTR): NTR1477

## Background

Depression is a common health problem in nursing home (NH) residents: prevalence rates vary from 6 to even 50% [1-3]. Depression is strongly related to quality of life of NH residents [4], it seriously impacts wellbeing and daily functioning, and increases use of health care services and even mortality [5-7]. The association between depression and quality of life highlights the importance of identifying and treating depression in NH residents with and those without dementia [8,4]. Unfortunately, although depression is a treatable disorder [9], various studies have shown poor detection and undertreatment of depression in NH residents [2,10-12].

Several studies have demonstrated effects of pharmacological and psychosocial interventions for depression in nursing homes [13,14]. The review of Bharucha et al. [15] of 'talk therapies' for depression in long-term care presents evidence for an improvement in depressive symptoms after reminiscence/life review therapy. Moreover, there is evidence for the effectiveness of multifaceted interventions in residential care [16-18] and in nursing homes [19,20].

The Nijmegen University NH Network (UKON), a collaboration between 12 care organizations and the Department of Primary and Community Care of the Radboud University Nijmegen Medical Centre, has developed the care program Act In case of Depression (AID), a multidisciplinary care program to identify and treat depression and monitor treatment effects. The care program is based on and in accordance with the recommendations as formulated in the Supplement Older Adults of the multidisciplinary evidence based guideline for diagnosis and treatment of depression [21] and the Consensus Statement of the American Geriatrics Society and the American Association of Geriatric Psychiatry [22]. The care program is an implementable plan of work that coordinates how the different disciplines should work together, fits in daily practice, and describes how new working methods are related to and can be integrated in the present care process following a step-by-step plan [23].

To date, cost effectiveness studies into the management of depression in NH have not been carried out, but are requested [24]. Gruber-Baldini et al. [10] did find increased involvement of mental health professionals in depressed long-term care residents with dementia, and Smalbrugge et al. [6] found that depressed residents of somatic units had increased use of medication, and received medical specialist consultation and treatment more often than non-depressed residents, implying expensive medical tests and hospital admissions. This paper describes a study that will evaluate the cost effectiveness of the care program AID.

## Methods/Design

The study is a stepped wedge, multicentre intervention study on 14 somatic and 14 Dementia Special Care (DSC) units of UKON-NH.

**Figure 1:**
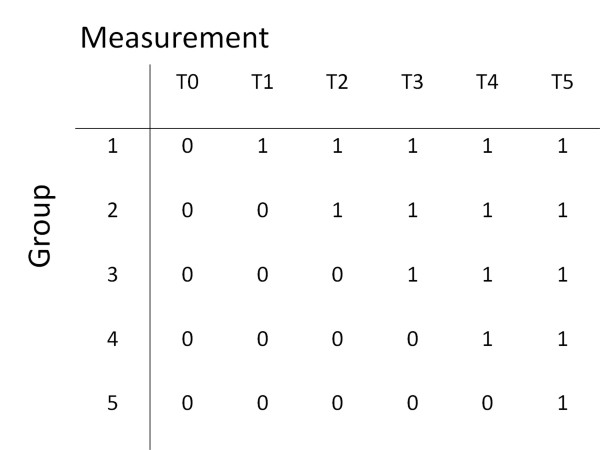
**Graphical representation of the stepped wedge design**. '0' represents measurement of usual care; control condition '1' represents measurement after the intervention has been implemented; intervention-condition

A stepped wedge design is a type of crossover design in which different clusters (here: units) cross over from the control group to the intervention group at different time points. All clusters are measured at each time point. The first time point corresponds to a baseline measurement where none of the clusters receive the intervention of interest; at the last time point all clusters receive the intervention. After intermediate time points, clusters initiate the intervention. More than one cluster may start the intervention at a time point, but the time a cluster begins the intervention is randomized [25] (see Figure 1 for a graphical representation of the design). This way, comparisons within units ánd between units will be available, making the design very powerful. Another advantage of the design is that all involved units will receive the intervention - which is expected to increase motivation for participating in the study.

At the start of the data collection, the residents with informed consent of all 28 units are screened for depression (T0). Following this, each of the units is randomly assigned to one of 5 groups. Each group starts the intervention at different time points, directly after one of the measurements (T0-T4), which are each 4 months apart. In the four- month interval between T0 and T1, nursing staff of the first group is trained within the first month. After this month, the intervention runs for the subsequent 3 months in the first group before the second measurement (T1) of all 28 units takes place. After T1, the second group is trained, and the intervention starts in this group while it is continued in the first group. This procedure is repeated for the remaining 3 groups until, at the last measurement (T5), all 28 units are in the intervention condition. Consequently, the follow up in the intervention condition varies from 3 months for the last group, which starts with the intervention 1 month after T4, to 19 months for the first group, which starts after T0.

### Intervention

**Figure 2:**
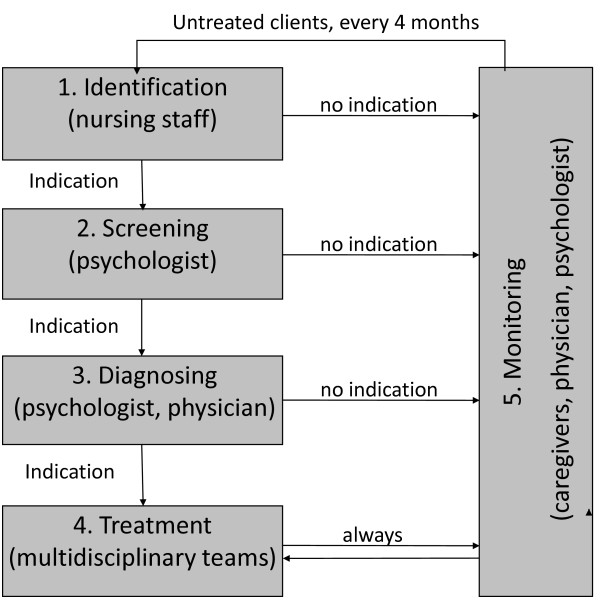
**Graphical representation of the AID care program**.

Figure 2 shows the care program AID. AID proposes an evidence and practice based standardization of 5 components in the management of depression: 1) identification of depressive symptoms, 2) screening, 3) diagnosis, 4) treatment and 5) monitoring. AID includes standardized use of measurement instruments and diagnostical methods, and protocolized treatment that combines psychosocial, psychological and pharmacological interventions. Cooperation between the disciplines is prearranged. As the ability of nursing staff to detect depression can and should be enhanced [26], the multifaceted and multidisciplinary care program 'AID' starts with a training program for nursing staff on how to identify symptoms of depression using a short observation scale [27] and how to support NH residents with depressive symptoms or depression. Further, AID comprises plans of work for the identification, screening, diagnosing, treatment and monitoring of depression.

#### Identification

nursing staff completes a short observation scale for depression [27] for all participating residents on the unit. If according to the scores on the scale further screening is indicated, nursing staff contacts the psychologist who takes over the coordination on the screening and diagnosing. If no further screening is indicated, nursing staff will complete the observation scale again after 3-4 months.

#### Screening

The psychologist screens the 'identified' residents of somatic units for depressive symptoms with the GDS-8 (Geriatric Depression Scale-NH version; cut-off score 2/3) [28] and those of DSC units with the CSDD (Cornell Scale for Depression in Dementia; cut-off score 7/8) [29-31]. If screening with the GDS-8 in somatic residents is problematic because of cognitive or communication problems, the CSDD will also be administered [32].

For residents with depressive symptoms, i.e. total scores on the GDS-8 or CSDD above the cut-off score, a diagnostic procedure will follow. For other residents, the identification phase will be repeated after 3-4 months.

#### Diagnosing

The elderly care physician and psychologist of each unit perform a diagnostic procedure including the use of chart information, caregiver interview, and examination of the resident (interview, physical examination). Diagnosis of major depression is established according to the DSM-IV-TR criteria. For minor depression the same criteria are used while only 2 to 4 symptoms are present [33,34]. In residents with dementia the Provisional Diagnostic Criteria for depression in Alzheimer's disease are applied (PDC) [35].

#### Treatment

Somatic and dementia residents with depressive symptoms, but without a clinical diagnosis of depression, are offered a personal day structure program made by the nursing staff in collaboration with the recreational therapist. Exercise and music therapy can be part of this day program. Psycho-education is also offered to the resident and/or relatives, including information about depressive symptoms and coping strategies.

Somatic residents with minor depression receive the same treatment as residents with depressive symptoms extended with individual life review therapy. This therapy is based on a protocol that has already been used successfully in Dutch residential care residents and is developed in close collaboration with the Dutch life review expert E. Bohlmeijer [36].

Somatic residents with major depressive disorder receive the same treatment as residents with minor depression extended with pharmacological treatment, when deemed appropriate by the elderly care physician. Prescription of pharmacological therapy is in accordance with the recommendations of the Supplement Older Adults [21].

For dementia residents with a PDC-depression diagnosis, treatment includes a personal day structure program, a behavioral management strategy developed by the psychologist and psycho education - especially of relatives. Apart from that, psychological treatment is offered: the clinical experts involved in the development of this care program agreed with recommendations made in the Supplement Older Adults [21] to intervene through the nursing staff (mediative therapy), but stressed that individual contact with the resident is also a necessity. Thus, for dementia residents, psychological treatment comprises of the psychologist supporting and supervising the nursing staff and recreational therapist more intensively in their execution of the day structure program and behavioral management strategy. This support takes place in a regular staff meeting, every two weeks. Within 1 month after the diagnosis, the day structure program and behavioral management strategy should be incorporated in regular care. The psychologist supervises the recreational therapist and nursing staff in at least 2 regular staff meetings. Additionally, if the depression in dementia residents is severe, pharmacological therapy can be given by the elderly care physician, when deemed appropriate.

#### Monitoring

Monitoring with a validated measurement instrument takes place to evaluate treatment. For this purpose, the GDS-8 is used in somatic residents, and the CSDD is used in dementia residents.

### Sampling

We calculated the sample-size using the following assumptions.

For somatic units: 25 residents per unit [37], a depression prevalence of 22% [38], a remission rate of 40% [39], and an attrition of 20% [38].

For DSC units: 20 residents per unit [40], a depression prevalence of 30% [10,40-42], a remission rate of 35% [13], and negligible attrition [40].

Based on these assumptions and a significance level alpha of 0.05, a power of 0.80 and an ICC of 0.1 for both somatic and dementia residents, 14 clusters (units) with 6 measurements are needed in a stepped wedge design to allow multilevel analysis.

Given that the outcomes will be presented on unit-level, during the data collection, newly admitted residents and/or their legal representatives are asked to provide informed consent on all units. This way, the sample size is not influenced by death or relocation of participating residents and can remain stable.

### Ethical approval

The Medical Ethics Committee of the Radboud University Nijmegen Medical Centre (CMO Arnhem-Nijmegen) rated the study and pronounced that it is not burdensome for the participant. Each NH resident and/or the legal representative on the participating units receives written and verbal information prior to the AID study and is only included in the study after having given written informed consent.

### Measurements

*Primary outcomes *are frequency of depression and quality of life.

Frequency of depression (the percentage of residents with depression on a unit) is measured in somatic residents by a shortened version of the Geriatric Depression Scale (GDS)[43], the 8-item GDS-nursing home version (GDS-8) of Jongenelis et al. [28], which was made by deleting GDS-items that are not applicable to most NH residents. The GDS-8 was validated in the AGED dataset, where it showed a good internal consistency of α = .80 and high sensitivity rates of 96.3% for major depression and 83.0% for minor depression, with a specificity rate of 71.7% at a cut-off score of 3 or more [28]. The GDS-8 also appears to be able to assess (change in) severity of depression [44]. The GDS-30 is originally a self-report instrument, the GDS-8 is interview based.

Frequency of depression in dementia residents is measured by the Cornell Scale for Depression in Dementia (CSDD)[29]. The CSDD is administered through interviewing nursing staff about their observations of the residents' behavior. The CSDD consists of 19 items each rated as 0 = absent, 1 = mild or intermittent and 2 = severe. The scores of the individual items are summed and a cut-off of 8 or more indicates depression [29]. Vida et al. [30] reported for a cut-off score of 8 or more, a sensitivity of 90% and specificity of 75% in residents with Alzheimer's Disease.

Quality of life in somatic residents is measured by the EQ-5D [45]. The EQ-5D instrument is a standardized non disease-specific instrument for describing and valuing Health Related Quality of Life [46]. There are two core components of the instrument: a description of the respondent's 'own health' using a health state classification system with five domains (mobility, self-care, usual activities, pain/discomfort and anxiety/depression) and a rating of 'own general health' by means of a visual analogue 'thermometer' scale. The EQ5D has shown a good validity and good test-retest reliability [47,48].

In dementia residents quality of life is measured by the EQ-5D proxy version [49]. Thereto, nursing staff are asked to score the scale for the resident.

Secondary outcomes are percentage accuracy of depression-detection in usual care, prevalence of depression-diagnosis in the intervention group, and response to treatment of depressed residents.

*Additional measurements *involve measurement of cognitive functioning by the Mini Mental State Examination (MMSE) [50] and measurement of sociodemographic variables, mental health history - including prior depressive episodes-, present mental health condition - including a dementia diagnosis -, possible treatment for depression, and somatic comorbidity.

Measurements are done by the research team. To study the compliance to the care program, the actual use of all components of the psychosocial, psychological and pharmacological treatment, as well as the factors determining this use, are registered. Accordingly, written checklists are used for nursing staff, recreational therapist, psychologist and elderly care physician, separately.

### Data-analysis

Primary effects will be calculated using multilevel regression analysis, for somatic and DSC units separately. The GDS-8-scores and CSDD-scores will be used in the primary analysis. Age, sex, cultural background and cognitive status will be used as covariates. The EQ5D will be analyzed as another primary outcome in the intervention study. For cost analysis, see economic evaluation. A process analysis will be carried out to determine the actual use of the components of the psychosocial, psychological and pharmacological treatment, and to determine facilitators and obstacles.

Secondary outcomes (percentage accuracy of depression-detection in usual care, prevalence of depression-diagnosis in the intervention group and response to treatment of depressed residents) will be analyzed using descriptive statistics.

### Economic evaluation

This study investigates the efficiency of the care program AID compared to usual care as provided in NH units. If the program AID turns out to be successful, a decrease in the prevalence of depression in NH will occur. On the one hand the program needs investment in for example training of nursing staff and, consequently, generates extra costs compared to usual care. On the other hand it potentially generates savings as it reduces depression related time investment in NH.

The economic evaluation is based on the general principles of a cost-effectiveness analysis from a healthcare viewpoint. Based on the above mentioned primary outcomes, two different incremental cost effectiveness ratios (ICERs) will be computed, answering the questions: 'How much money has to be invested additionally in the care program to gain one percentage point decrease in frequency of depression?' and 'How much money has to be invested additionally in the care program to gain one Quality-Adjusted Life Year (QALY)?'

The cost analysis consists of two main parts. First, on resident level, volumes of care (to determine the incremental direct health care costs) based on the production process of the care program and of depression decrease are measured prospectively using an activity based costing approach. Focusing on activities performed with costs accumulated at the activity level(s) of the health care production processes, standardized case report forms are used to assess time invested by nursing staff, psychologist, elderly care physician and recreational therapist. Also, number of hospital admissions (number of days in hospital) and use of antidepressant medication are recorded.

Second, the cost prices for each volume of consumption will be determined to use these for multiplying the volumes registered for each participating resident. The Dutch guidelines for cost analyses will be used [51]. For units of care/resources where no guideline or standard prices are available, real cost prices will be determined. Statistics of the total costs per resident will be determined for usual care and care according to the care program AID. Depending on the skewness of the parameter distributions, statistical testing of differences between strategies will be of a parametrical or non parametrical nature. The impact of deterministic variables, such as cost prices for volume parameters that are incremental cost drivers will be investigated using sensitivity analyses on the basis of the range of extremes.

The effect analysis adheres to the design of the study. Relevant for the economic evaluation are the frequency of depression (measured with GDS-8 and CSDD) and QALYs (utilities measured with the EQ-5D). Using the trapezium rule, the QALYs will be computed in order to perform a cost-effectiveness analysis comparing the two alternative strategies. Change in utilities (EQ-5D) will be based on the mean values for the residents when they are in the control condition and the mean values after having been in the intervention for 3 (all 5 groups), 7 (4 groups), 11 (3 groups), 15 (2 groups) and 19 months (1 group). ICERs will be computed and sampling uncertainty will be determined using the bootstrap or Fieller method. Finally, a cost-effectiveness acceptability curve will be derived that is able to evaluate efficiency by different thresholds for the ICERs.

## Discussion

In this paper we described the design of a randomized trial to evaluate the (cost-)effectiveness of a multidisciplinary, evidence based care program to improve the management of depression in NH residents of somatic and DSC units. This study holds several unique elements.

First of all, the Department of Primary and Community Medicine of the Radboud University Nijmegen Medical Centre has established a structural collaboration with 12 care organizations (representing 40 NH and 100 residential homes) in the Nijmegen University NH Network (UKON). An expert group of the UKON has developed the care program AID, based on evidence based guidelines and the Consensus Statements [21,22]. Implementation is expected to be successful, because it fits with daily practice and describes how new working methods are related to and can be integrated in the present care process following a step-by-step plan [23].

Secondly, the intervention is based on a stepped care approach: the more serious the depressive complaints or the depression, the more intense the intervention will be. The standardized interventions will be tailored to the needs of the individual resident. This will expectedly increase its effectiveness and facilitate transferring this strategy to other nursing homes.

Finally, the design of the study -the stepped wedge design- is a relatively new design, and has not been applied before in long term care. Using a stepped wedge design signifies that all participating units will cross-over from the control condition to the intervention condition during the study. This is expected to increase the motivation of NH workers to participate in scientific research.

In conclusion, the care program is expected to be effective in reducing the frequency of depression and in increasing the quality of life of residents. The study also will provide insight in the program's cost- effectiveness.

## Competing interests

The authors declare that they have no competing interests.

## Authors' contributions

DLG designed the study and the intervention, and wrote the paper. MS designed the study and the intervention, and co-wrote the paper. ST introduced and planned the stepped wedge design of the study. RL co-designed the intervention and co-wrote the paper. EA designed the economic evaluation of the study. MVD assisted in the design of the study and co-wrote the paper. ED assisted in the design of the study and the intervention and co-wrote the paper. RK assisted in the design of the study and co-wrote the paper. All authors read and approved this manuscript.

## Pre-publication history

The pre-publication history for this paper can be accessed here:

http://www.biomedcentral.com/1471-244X/11/91/prepub
